# Supplementary material for: The causal role of circulating inflammatory markers in osteoporosis: a bidirectional Mendelian randomized study
Source: Front Immunol. 2024 Jul 18;15:1412298. doi: 10.3389/fimmu.2024.1412298 (PMC11291241; doi:10.3389/fimmu.2024.1412298)
Supplement: Supplementary file 1 [file DataSheet_1.zip › supplementary material/CIM-OSTEOPOROSIS /GCST90274804.csv_forest.pdf]

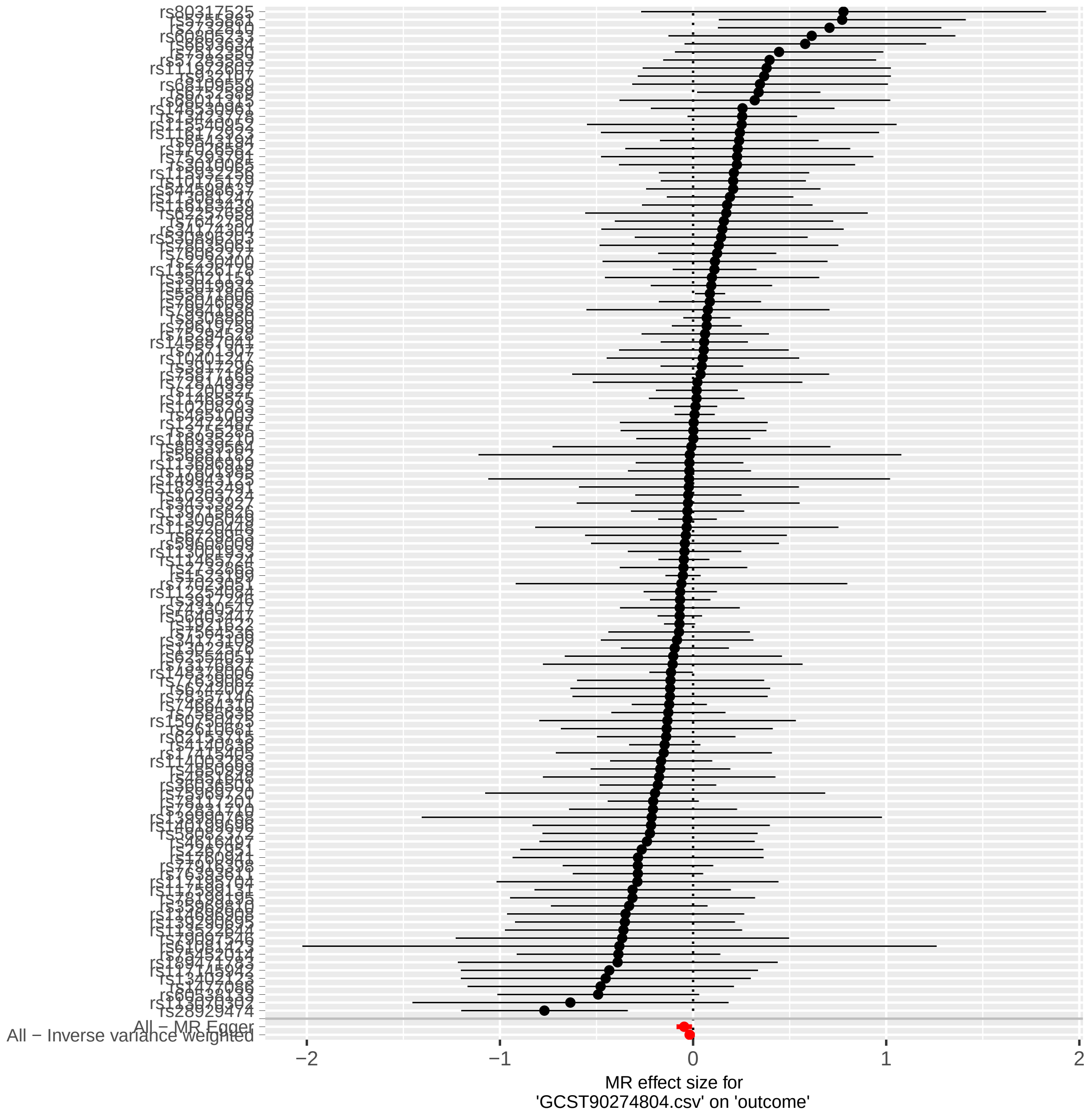
rs80317525
rs28929474
All − MR Egger
All − Inverse variance weighted
−2
−1
0
1
2
MR effect size for
'GCST90274804.csv' on 'outcome'
